# Supplementary material for: Chronic intracranial recordings in the globus pallidus reveal circadian rhythms in Parkinson’s disease
Source: Nat Commun. 2024 May 30;15:4602. doi: 10.1038/s41467-024-48732-0 (PMC11139908; doi:10.1038/s41467-024-48732-0)
Supplement: Supplementary file 1 — Supplementary Information [file 41467_2024_48732_MOESM1_ESM.pdf]

Supplementary Information

Supplementary Note

The STN and GPi are the main targets for DBS therapy in PD. Due to the clinical decision-making process at UF, far fewer patients were implanted in the STN compared to the GPi. Based on the same inclusion criteria as the main GPi cohort, only 23 subjects with PD (30 unique hemispheres) implanted with electrodes in STN in the UF INFORM Database were eligible for analysis. The STN subjects' demographic information is presented in **Supplementary Table 1**.

The STN dataset was analyzed using the same methods and statistical analysis used for the GPi dataset. All methods and analyses are described in the main manuscript.

STN power mainly decreases at nighttime but may sometimes increase

Supplementary Figure 1 shows two individual examples with STN power decreased at night (**A**) and increased at nighttime (**B**), with the corresponding polar plots showing the distribution of STN power and time of events as well as the normalized power averaged over 24h. For group analyses, the neural power was z-score normalized and averaged over daytime (3:00-8:00 pm) and nighttime (0:00-5:00 am). **Panel 1C** shows each individual STN recording (N=30 hemispheres), normalized, averaged, and plotted over 24h, sorted by the highest power in the daytime, and grouped in the main canonical spectral band (alpha, low beta, and high beta). Visual inspection shows the presence of a strong circadian rhythm in most recordings. We found increased power at nighttime in 20% of all recordings (6 hemispheres) while decreased power was observed in 70%% (21 hemispheres). In 3 hemispheres (10%), there was no statistically significant difference between daytime and nighttime power distributions (t-test, $p_{corrected}>0.05$). When analyzing the beta band (13-30 Hz) separately, we found that 71% (17 hemispheres) showed a decreased in power at nighttime, 17% (4 hemispheres) showed an increased in power at nighttime, and 12% (3 hemispheres) of the recordings had no statistically significant difference in power between daytime and nighttime (t-test, $p_{corrected}>0.05$). The proportion of patients with increased beta power at nighttime was similar to that of the GPi subjects (16% vs 17.0%).

STN circadian rhythms differ across frequency bands

Supplementary Figure 2 shows complementary statistical analyses for STN circadian rhythms. **Panel 2A** shows the distribution of changes in neural power between daytime and nighttime based on the center sensing frequency. When analyzing the relationship between sensing frequency and circadian rhythms, one-way ANOVA revealed a significant effect of sensing frequency on the difference in power between day and night

(F=3.68, $p_{corrected}$=0.04). Post-hoc analysis with Tukey's test showed that the circadian rhythms in the high beta band showed more decreased power at nighttime than low beta (q=-0.95, $p_{corrected}$=0.05) and alpha (q=-1.14, $p_{corrected}$=0.05) (**Supplementary Figure 2B**). Parametric tests within each canonical frequency showed that high beta band power was decreased more at nighttime (t=9.09, $p_{corrected}$=0.002,). All p values were corrected for multiple comparison with Bonferroni correction. t-test

STN circadian rhythms are not affected by other factors

Similar to GPi analysis, a general linear model (GLM) was built to test the effects of stimulation parameters, medications, and motor phenotype on the beta power (13-30 Hz) circadian rhythms in STN. In particular, we investigated whether the change in beta power (dependent variable) between daytime and nighttime was significantly modulated by the following independent variables: the levodopa equivalent daily dose (LEDD)[22,23], the use of extended-release levodopa at nighttime, the use of non-dopaminergic medication at nighttime, the location of contact used for stimulation (ventral vs dorsal), the total electrical energy delivered (TEED), the Unified Parkinson's disease rating scale (UPDRS) total scores, and the subject's motor phenotype (tremor, intermediate, or akinetic subtype). The PD motor phenotype, LEDD, and TEED were calculated based on established methods[22–25]. The contact used for stimulation was defined as ventral (stimulation contact 1) or dorsal (stimulation contact 2). No significant contributor to beta band circadian rhythms was found among all variables included in the analysis (**Supplementary Table 2**). However, this may be due to the low sample size. It should also be noted that none of the STN subjects used nighttime extended-release levodopa, which was the primary contributor to the beta band circadian rhythms in the GPi.

Finally, when comparing STN beta activity recorded in the awake state off-medication and off-stimulation (baseline beta power) in subjects with increased power at nighttime and decreased power at nighttime, no statistically significant difference was found (U=51, $p_{corrected}$=0.4, **Supplementary Figure 2C**).

Supplementary Table 1. STN subject demographics, clinical characteristics, and recording settings.

	STN
DEMOGRAPHICS	
**TOTAL NUMBER OF SUBJECTS**	23 (30 hemispheres)
**SEX**	M: 16 (69.6%) F: 7 (30.4%)
**AGE AT SURGERY (YEARS)**	66.2 ± 7.0
**DISEASE DURATION (YEARS)**	12.3 ± 8.8
SYMPTOMS AND THERAPY CHARACTERISTICS	
**PREOPERATIVE UPDRS**	38.2 ± 13.1
**TEED (μJ)**	93 ± 80
**LEDD (MG)**	1033 ± 486
RECORDING SETTINGS	
**RECORDING DURATIONS (DAYS)**	5 ± 0
**TIME SINCE IMPLANT (DAYS)**	35.3 ± 65.5
**SENSING FREQUENCY (HZ)**	16.2 ± 3.9

Supplementary Table 2. Generalized linear model for the effect of stimulation therapy, medications, and motor symptoms on beta band (13-30 Hz) circadian rhythms.

	Coefficient	Std. Err.	z	P>|z|
Intercept Term	0.9598	0.468	2.05	0.04
**Stimulation Location (Dorsal: 1, Ventral: 0)**	-0.0782	0.357	-0.219	0.827
**TEED (μJ)**	2242.634	1935.576	1.159	0.247
**LEDD (mg)**	-4.00E-04	0	-1.228	0.219
**Nighttime Levodopa ER (Use: 1, Not Used: 0)**	3.37E-11	2.91E-11	1.159	0.247
**Sleep Medication (Used: 1, Not Used: 0)**	-0.4902	0.419	-1.169	0.242
**PD Subtype (Tremor/Akinetic Ratio)**	-1.5078	2.057	-0.733	0.464
**UPDRS**	0.0526	0.058	0.901	0.367

ER: Extended-release, TEED: total electrical energy delivered, LEDD: levodopa equivalent daily dosage, UPDRS: Unified Parkinson's Disease Rating Scale, μJ: microjoule, mg: milligram. Source data is provided in the Source Data file

Figures

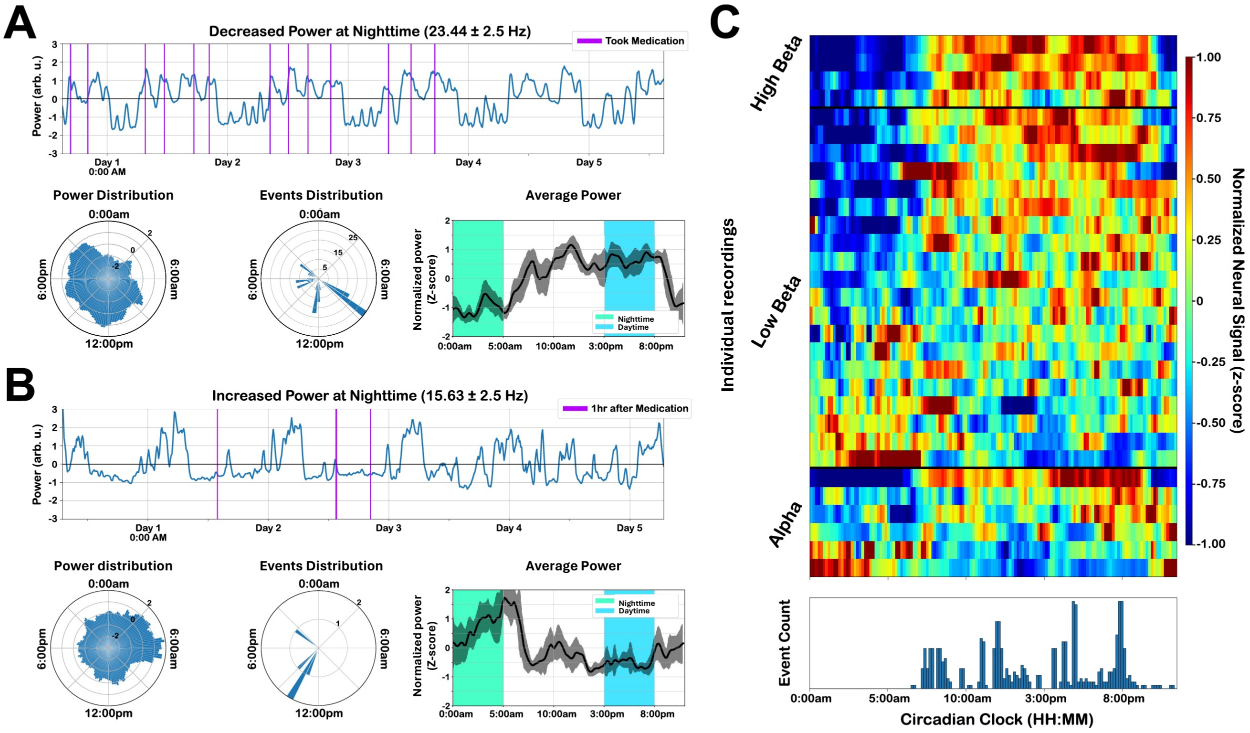

Supplementary Figure 1. Circadian rhythms in the STN. A) Example of a subject showing STN beta band (23.44 ± 2.5Hz) decreasing at night. Pink vertical lines indicate the time of events marked by the subject and are used here as a surrogate marker of the asleep/awake period. Circular polar plots show the beta power (left panel) and the number of events marked (middle panel) in a 24-hour circadian clock with a 1-hour averaged increment. The mean and standard error of the normalized spectral power averaged over a 24-hour cycle is shown on the right panel. The green and blue shades indicate the nighttime and daytime periods, respectively, used for subsequent analysis (see methods). **B**) Example of a subject showing STN beta band (15.63 ± 2.5Hz) increasing at night. Same convention as in panel A. **C**) Circadian heatmap showing STN power from all individual recordings (n=30) normalized and plotted over a 24-hour circadian clock. STN power is sorted from the most decreased (blue) to the most increased (red) at night. The number of events reported by all subjects over the 24-hour circadian clock is shown below the circadian heatmap and used as a surrogate of the awake/sleep period. Hz: Hertz; arb.u.: arbitrary units; Source data is provided in the Source Data file.

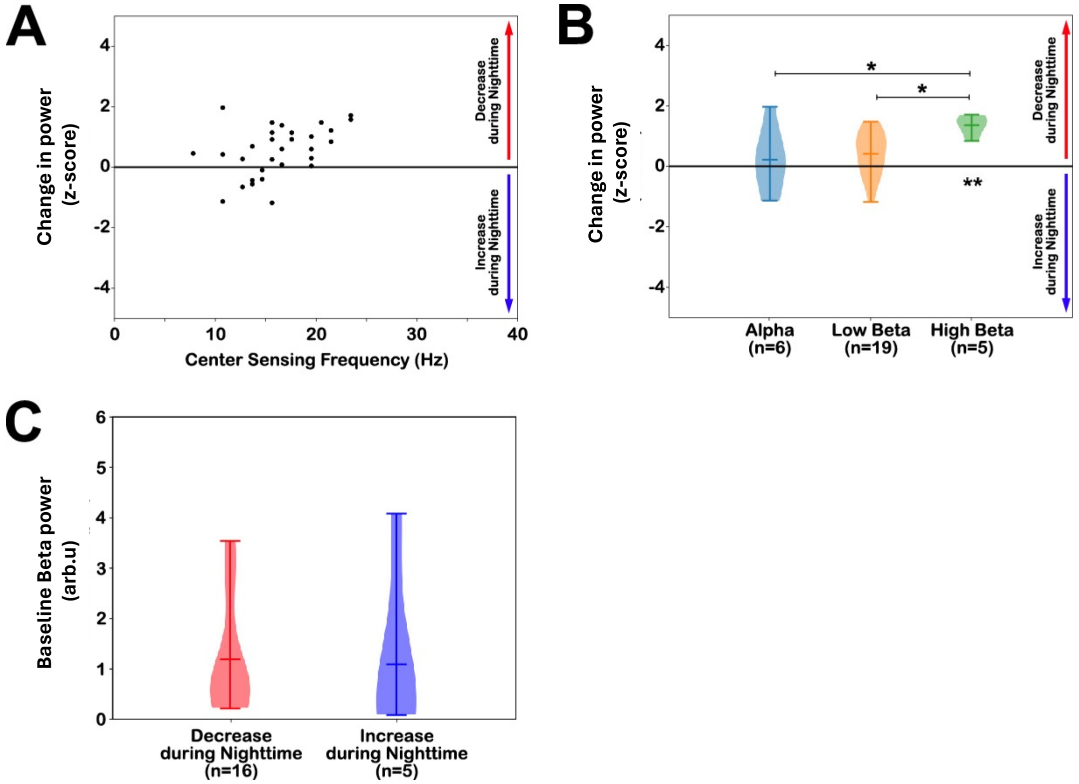

Supplementary Figure 2. Factors influencing circadian rhythms in the STN. A) Scatter plot of the changes in spectral power between daytime and nighttime period (Day – Night), for each STN hemisphere recording (n=30), in relation to the center frequency of the recordings. A positive change indicates a decrease in power at nighttime and a negative change indicates an increase in power at nighttime. **B**) Violin plot quantifying the changes in power during day and night for each canonical frequency band (mean and range); alpha (<=12 Hz), low beta (>12 and <=20 Hz), and high beta (>20 and <=30 Hz). High beta ($p_{corrected}$=0.002) distributions had more decreased power at nighttime (indicated by positive values and ** below violins). However, alpha and low beta were not statistically different from 0 ($p_{corrected}$=1.0 and $p_{corrected}$=0.06, respectively), indicating that power was either increased or decreased at night. The high beta band was statistically different from the alpha band ($p_{corrected}$=0.05) and the low beta band ($p_{corrected}$=0.05) using post-hoc Tukey's tests following a one-way ANOVA. **C)** Comparison of in-clinic off-medication off-stimulation awake baseline beta band power in subjects with increased and decreased power at nighttime. There was no statistical difference between the two groups ($p_{corrected}$=0.36). (*) denotes p < 0.05, (**) denotes p < 0.01, and (***) denotes p < 0.001. All p values were corrected for multiple comparisons with Bonferroni correction. Hz: Hertz; arb.u.: arbitrary units; n: number of recordings. Source data is provided in the Source Data file.
