# Supplementary material for: Chronic intracranial recordings in the globus pallidus reveal circadian rhythms in Parkinson’s disease
Source: Nat Commun. 2024 May 30;15:4602. doi: 10.1038/s41467-024-48732-0 (PMC11139908; doi:10.1038/s41467-024-48732-0)
Supplement: Supplementary file 3 — Reporting Summary [file 41467_2024_48732_MOESM3_ESM.pdf]

nature portfolio

Corresponding author(s): Coralie de Hemptinne
Last updated by author(s): 4/12/2024

Reporting Summary

Nature Portfolio wishes to improve the reproducibility of the work that we publish. This form provides structure for consistency and transparency in reporting. For further information on Nature Portfolio policies, see our Editorial Policies and the Editorial Policy Checklist.

Statistics

For all statistical analyses, confirm that the following items are present in the figure legend, table legend, main text, or Methods section.

n/a	Confirmed	
☐	☒	The exact sample size (*n*) for each experimental group/condition, given as a discrete number and unit of measurement
☐	☒	A statement on whether measurements were taken from distinct samples or whether the same sample was measured repeatedly
☐	☒	The statistical test(s) used AND whether they are one- or two-sided<br>*Only common tests should be described solely by name; describe more complex techniques in the Methods section.*
☐	☒	A description of all covariates tested
☐	☒	A description of any assumptions or corrections, such as tests of normality and adjustment for multiple comparisons
☐	☒	A full description of the statistical parameters including central tendency (e.g. means) or other basic estimates (e.g. regression coefficient) AND variation (e.g. standard deviation) or associated estimates of uncertainty (e.g. confidence intervals)
☐	☒	For null hypothesis testing, the test statistic (e.g. *F*, *t*, *r*) with confidence intervals, effect sizes, degrees of freedom and *P* value noted<br>*Give P values as exact values whenever suitable.*
☒	☐	For Bayesian analysis, information on the choice of priors and Markov chain Monte Carlo settings
☒	☐	For hierarchical and complex designs, identification of the appropriate level for tests and full reporting of outcomes
☒	☐	Estimates of effect sizes (e.g. Cohen's *d*, Pearson's *r*), indicating how they were calculated

Our web collection on statistics for biologists contains articles on many of the points above.

Software and code

Policy information about availability of computer code

Data collection	For this retrospective study, data collection was done via the UF INFORM database, a centralized database with clinical-demographic data extracted from the Epic EMR, preoperative and postoperative imaging as well as brain signal recordings. Images were processed using Advanced Normalization Tools (Ants) and 3D slicer software.
Data analysis	All analyses were performed using custom script in Python 3.10<br>Data extraction and visualization was done using The Brain Recording Analysis and Visualization Online (BRAVO) Platform v2.2.0 developed at University of Florida and shared as an open source tool (Cagle et. al., 2023, Brain Stimul). The analysis codes generated in this study are available at https://github.com/Fixel-Institute/Publication-Scripts.

For manuscripts utilizing custom algorithms or software that are central to the research but not yet described in published literature, software must be made available to editors and reviewers. We strongly encourage code deposition in a community repository (e.g. GitHub). See the Nature Portfolio guidelines for submitting code & software for further information.

Data

Policy information about availability of data

All manuscripts must include a data availability statement. This statement should provide the following information, where applicable:

- Accession codes, unique identifiers, or web links for publicly available datasets
- A description of any restrictions on data availability
- For clinical datasets or third party data, please ensure that the statement adheres to our policy

All data supporting the findings of this study are available within the article and its Supplementary files. Any additional requests for information can be directed to, and will be fulfilled by, the corresponding authors. The raw (identifiable) data from participants are privacy-protected. The processed de-identified data will be shared upon request as these datasets are part of ongoing research study. Source data are provided with this paper.

Research involving human participants, their data, or biological material

Policy information about studies with human participants or human data. See also policy information about sex, gender (identity/presentation), and sexual orientation and race, ethnicity and racism.

Reporting on sex and gender	This retrospective study includes 93 PD patients from the UF INFORM database who met the inclusion criteria. Of these, 72 were males and 21 were females (77.4% and 22.6% respectively). There were no sex or gender specific analysis other than demographic information of our patient population.
Reporting on race, ethnicity, or other socially relevant groupings	There were no race or ethnicity specific analysis because these informations are not available in retrospective data collection.
Population characteristics	This retrospective study includes 93 PD patients (130 unique hemispheres with recordings) from the UF INFORM database who met the inclusion criteria. Of these, 72 were males and 21 were females (77.4% and 22.6% respectively), with an average age at surgery of 68.3 ± 9.4 (mean ± std) years and an average disease duration of 14.1 ± 5.9 (mean ± std) years. Subjects were implanted with the Medtronic Percept PC neurostimulator attached to unilateral (n = 38, 40.9%) or bilateral (n = 55, 59.1%) DBS leads in the GPi. Among them, 58 hemispheres (44.6%) were implanted with Medtronic 3387 quadripolar DBS electrodes, and 72 hemispheres (55.4%) were implanted with Medtronic SenSight segmented DBS electrodes.
Recruitment	This is retrospective data collection. All patients who met the inclusion criteria of this study were included in this dataset and analyses
Ethics oversight	This retrospective study was performed at the Norman Fixel Institute for Neurological Diseases at the University of Florida (UF). All subjects provided informed consent in accordance with the Declaration of Helsinki to participate in the UF INFORM Database, a database that stores participants' clinical records, imaging and neural recordings (IRB# 201501166). This study was approved by the UF Institutional Review Board (IRB) to request and process data from the UF INFORM Database (IRB# IRB202202094).

Note that full information on the approval of the study protocol must also be provided in the manuscript.

Field-specific reporting

Please select the one below that is the best fit for your research. If you are not sure, read the appropriate sections before making your selection.

[x] Life sciences [ ] Behavioural & social sciences [ ] Ecological, evolutionary & environmental sciences

For a reference copy of the document with all sections, see nature.com/documents/nr-reporting-summary-flat.pdf

Life sciences study design

All studies must disclose on these points even when the disclosure is negative.

Sample size	This retrospective study was performed at the Norman Fixel Institute for Neurological Diseases at the University of Florida (UF). All data from the data that met the inclusion criteria was collected and analyzed without apriori ample size calculation.<br><br>Among the 347 PD patients with the Percept neurostimulator and who have consented to be part of the UF INFORM Database, 93 subjects (130 unique hemispheres with recordings) meet the inclusion criteria for this study. The inclusion criteria for this retrospective study were:<br>- individuals diagnosed with PD by a movement disorders-trained neurologist<br>- individuals implanted with unilateral or bilateral electrodes placed in the GPi and attached to the Medtronic Percept PC.<br>- individuals with neuronal power recorded for at least 5 consecutive days.<br>- individuals with DBS programmed in a monopolar stimulation (stimulation contact 1 or 2), allowing sensing from electrodes surrounding the stimulation contact to reduce stimulation artifacts.<br>-stable sensing and stimulation settings during the recordings
Data exclusions	The exclusion criteria were individuals diagnosed who did not have: a diagnosed of PD by a movement disorders-trained neurologist, who

were not implanted with DBS electrodes placed in the GPi and attached to the Medtronic Percept PC, or who did not have at least 5 consecutive days of chronic neural recording with stable stimulation.

Replication	This is a retrospective study and no perspective replication were done.
Randomization	This retrospective study was not a clinical trial and therefore randomization was not done.
Blinding	This study was observational and unblinded. However, we used the time of the 'events marked' as a surrogate of 'awake' time

Reporting for specific materials, systems and methods

We require information from authors about some types of materials, experimental systems and methods used in many studies. Here, indicate whether each material, system or method listed is relevant to your study. If you are not sure if a list item applies to your research, read the appropriate section before selecting a response.

Materials & experimental systems

n/a	Involved in the study
☒	☐ Antibodies
☒	☐ Eukaryotic cell lines
☒	☐ Palaeontology and archaeology
☒	☐ Animals and other organisms
☒	☐ Clinical data
☒	☐ Dual use research of concern
☒	☐ Plants

Methods

n/a	Involved in the study
☒	☐ ChIP-seq
☒	☐ Flow cytometry
☐	☒ MRI-based neuroimaging

Plants

Seed stocks	No Plant used
Novel plant genotypes	No Plant used
Authentication	No Plant used

Magnetic resonance imaging

Experimental design

Design type	No experiment
Design specifications	No experiment
Behavioral performance measures	No experiment

Acquisition

Imaging type(s)	Structural
Field strength	3T
Sequence & imaging parameters	Sagittal plane T1 MPRAGE Isometric
Area of acquisition	Whole Brain

Diffusion MRI ☐ Used ☒ Not used

Preprocessing

Preprocessing software	No preprocessing done

Normalization	Rigid + SyN 2 stage non-linear normalization done in Advanced Normalization Tools (ANTs).
Normalization template	ICBM 152 Asymmetric 2009b
Noise and artifact removal	Not performed.
Volume censoring	Not performed.

Statistical modeling & inference

Model type and settings	No modeling or inference done
Effect(s) tested	No modeling or inference done

Specify type of analysis: [x] Whole brain [ ] ROI-based [ ] Both

Statistic type for inference (See Eklund et al. 2016)	No modeling or inference done
Correction	No modeling or inference done

Models & analysis

n/a	Involved in the study
[x]	[ ] Functional and/or effective connectivity
[x]	[ ] Graph analysis
[x]	[ ] Multivariate modeling or predictive analysis
